# Trimodality PET/CT/MRI and Radiotherapy: A Mini-Review

**DOI:** 10.3389/fonc.2020.614008

**Published:** 2021-02-04

**Authors:** Pierre Decazes, Pauline Hinault, Ovidiu Veresezan, Sébastien Thureau, Pierrick Gouel, Pierre Vera

**Affiliations:** ^1^ Nuclear Medicine Department, Henri Becquerel Cancer Center, Rouen, France; ^2^ QuantIF-LITIS EA4108, University of Rouen, Rouen, France; ^3^ Radiotherapy Department, Henri Becquerel Cancer Center, Rouen, France

**Keywords:** computed tomography, magnetic resonance imaging, positron emission tomography, radiotherapy, hybrid imaging

## Abstract

Computed tomography (CT) has revolutionized external radiotherapy by making it possible to visualize and segment the tumors and the organs at risk in a three-dimensional way. However, if CT is a now a standard, it presents some limitations, notably concerning tumor characterization and delineation. Its association with functional and anatomical images, that are positron emission tomography (PET) and magnetic resonance imaging (MRI), surpasses its limits. This association can be in the form of a trimodality PET/CT/MRI. The objective of this mini-review is to describe the process of performing this PET/CT/MRI trimodality for radiotherapy and its potential clinical applications. Trimodality can be performed in two ways, either a PET/MRI fused to a planning CT (possibly with a pseudo-CT generated from the MRI for the planning), or a PET/CT fused to an MRI and then registered to a planning CT (possibly the CT of PET/CT if calibrated for radiotherapy). These examinations should be performed in the treatment position, and in the second case, a patient transfer system can be used between the PET/CT and MRI to limit movement. If trimodality requires adapted equipment, notably compatible MRI equipment with high-performance dedicated coils, it allows the advantages of the three techniques to be combined with a synergistic effect while limiting their disadvantages when carried out separately. Trimodality is already possible in clinical routine and can have a high clinical impact and good inter-observer agreement, notably for head and neck cancers, brain tumor, prostate cancer, cervical cancer.

## Introduction

External radiotherapy consists of treating an internal lesion, superficial and/or external lesion with an external source of radiation. In the nineties, computed tomography (CT) has revolutionized external radiotherapy by making it possible to visualize the tumor(s), corresponding to the target volume, and the organs at risk (OARs), which are normal tissues whose sensitivity to radiation can significantly influence treatment planning and/or prescribed dose. Because the treatment beams can be afterwards individually oriented on the tumor in a three-dimensional (3D) approach, this marked the beginning of 3D conformal radiotherapy (1).

A following improvement was the control of the intensity of the treatment beams which opened in the new millennium the era of the intensity-modulated radiotherapy (IMRT) whose aim is to deliver a high dose to the target volume while sparing the adjacent tissues, notably OARs (2). With this technique, a homogeneous dose is prescribed to the planning target volume (PTV) that considers uncertainty in treatment planning by encompassing the gross tumor volume (GTV, corresponding to the delineated macroscopic and radiologically measurable tumor) and the clinical target volume (CTV, which adds a margin to the GTV to cover nearby areas at risk of hosting microscopic disease) (3). The accuracy of anatomical localization is of particular importance for stereotactic radiotherapy (SRT), corresponding to an external beam radiotherapy used to deliver a high dose of radiation very precisely, as a single dose or a small number of fraction (4).

However, if CT imaging is a now a standard for radiotherapy, it presents some limitations, notably concerning tumor delineation which can be difficult, especially for soft tissues (3) or for the characterization of the lesions. Other 3D imaging modalities have therefore emerged for radiotherapy, in particular, positron emission tomography (PET) and magnetic resonance imaging (IRM). PET and MRI can notably visualize biological processes distinct and complementary to purely anatomical imaging. This led to the concept of biological target volume (BTV) focused on a metabolic function. For example, a boost radiotherapy can be performed on hypoxic tumors more resistant to radiation, identified by ^18^F-fluoromisonidazole (FMISO) PET/CT (5). Finally, CT, PET, and MRI can be used to follow the patient during the radiotherapy, at the end of the treatment, or during the follow-up (6)

If these 3D imaging modalities (CT, PET, and MRI) can be considered separately, they can also be associated to form a hybrid imaging, two by two (PET/CT, CT/MRI, PET/MRI) but also as a trimodality PET/CT/MRI. Multimodality is already possible in clinical routine with a high clinical impact and good inter-observer agreement (7).

The aim of this mini-review is to present the concept of PET/CT/MRI trimodality, its rationale for radiotherapy, and its potential interest in characterizing tumor, performing treatment planning, and doing ART.

## Technical Parts

### CT, MRI, and PET

CT is the reference imaging used by radiation oncologists for target volumes and organs at risk delineation for radiotherapy treatment planning. It is a high spatial resolution imaging modality that provides anatomical information with good spatial accuracy which is unaffected by geometric distortions. CT also provides a mapping of tissue electron density necessary for dosimetric calculations in radiotherapy. However, CT is an irradiating imaging modality with certain disadvantages such as lack of contrast in soft tissue and artifacts due to the presence of metal (8).

MRI is an anatomical and functional imaging modality that provides very good soft tissue contrast with millimetric spatial resolution. Although it has the advantage of being non-irradiating, the acquisition process is time consuming and this technique presents many contraindications (9). The possibility of using only MRI for radiotherapy treatment planning is however limited by the absence of information on tissue electronic density, a non-constant intensity of the images, and the presence of geometric distortions that deform images, including the volumes of interest.

CT and MRI are often associated with PET, a functional imaging modality. It provides a very good tumor/node contrast and the possibility to acquire large field of view. However, it is an irradiating examination with poor spatial resolution. In addition, the presence of partial volume artifacts creates blurred edges making more difficult the segmentation of volumes of interest.

Therefore, trimodality appears to be a technique of choice in the treatment of cancer in radiotherapy. It provides anatomical and functional information of high spatial resolution and allows improving the definition of target volumes in radiotherapy (10–12). A summary of the advantages and disadvantages of CT, MRI, and PET separately and combined in PET/CT/MRI is presented in **Table 1**.

**Table 1:** summary of the advantages and disadvantages of CT, MRI and PET separately and combined in PET/CT/MRI.

	CT	MRI	PET	Trimodality PET/CT/MRI
Advantages	Anatomy Spatial resolution (1 mm) Fast acquisition	Anatomy and function Spatial resolution (1 mm) Contrast (soft tissue) Non-irradiating	Function Tumor/Background Contrast Acquisition field	Anatomy and function Spatial resolution Tumor characterization Assessment of disease spread Dosimetry for RT
Limitations	Irradiating Contrast Artefacts (metal, teeth, *etc*.)	Long acquisition Compatible MRI equipment with high-performance dedicated coils Contraindications Artefacts (Distortions, no uniformity, *etc*.)	Irradiating Spatial resolution (>3–4 mm) Partial volume (blurred edges)	Irradiating Long acquisition Image registration Compatible MRI equipment with high-performance dedicated coils Contraindications MRI

### Trimodality PET/CT/MRI

As trimodality allows obtaining additional information on disease and tumors; images of each modality must be performed in radiotherapy treatment position (13). Each machine is equipped with a rigid table that is positioned on the device table. Each of these rigid tables has markers and an indexing system that allows fixing radiotherapy immobilization solutions. In order to have exactly the same position for all acquisitions, it is essential that the equipment used is compatible with all the installations and in particular non-magnetic equipment for MR. Patient repositioning on each device is done using markers on the skin (*i.e.* the positioning referential) made during the planning CT and external positioning lasers.

Currently, no medical device allows simultaneous acquisition of all three imaging modalities. The solution is to use two separate imaging devices, a bimodal hybrid machine and an independent machine. Two trimodality systems are possible: a PET/MRI coupled with a CT or a PET/CT coupled with an MRI (14). For each of them, image registration will be indispensable to delineate volumes of interest and to perform radiotherapy treatment planning (15).

For the solution with PET/MR, precautions must be taken for data acquisition and processing. The patient is positioned on the device with MR coils compatible with radiotherapy immobilization fixations (16). To perform attenuation correction on the PET image, an attenuation mapping of MR coils must be performed before (17). The PET/MR images and the planning CT are then registered before volume delineation and dosimetric planning. An alternative to this solution is to replace the planning CT by a synthetic CT, commonly referred as pseudo-CT (18, 19). With the emergence of artificial intelligence, new robust algorithms such as GANs (Generative Adversial Networks) (20, 21) allow the creation of attenuation maps, synthetic CT, from the different MR images. Treatment planning can then be performed without proceeding to the image registration step.

The second PET/CT + MRI solution is performed following the same process; the patient has these two examinations one after the other in the radiotherapy treatment position. Two techniques can be used for this PET/CT + MRI workflow. The first is to use a transfer system compatible with both imaging devices (22). This consists of an air cushion bed with low attenuation and a non-magnetic stretcher that allows the bed to be moved from one device to the other without moving the patient. The air-cushioned bed is placed on the rigid tabletop of the first imaging device, and the patient is positioned in a position in agreement with the positioning referential realized for planning CT. At the end of the acquisition, the patient on the air-cushioned bed is moved to the stretcher with the help of a suction system and is then transferred to the second imaging unit from the stretcher to the examination table using the same suction system. In the end, the system allows the realization of multi-modal acquisitions while keeping the patient in the same position.

**Figure 1:**
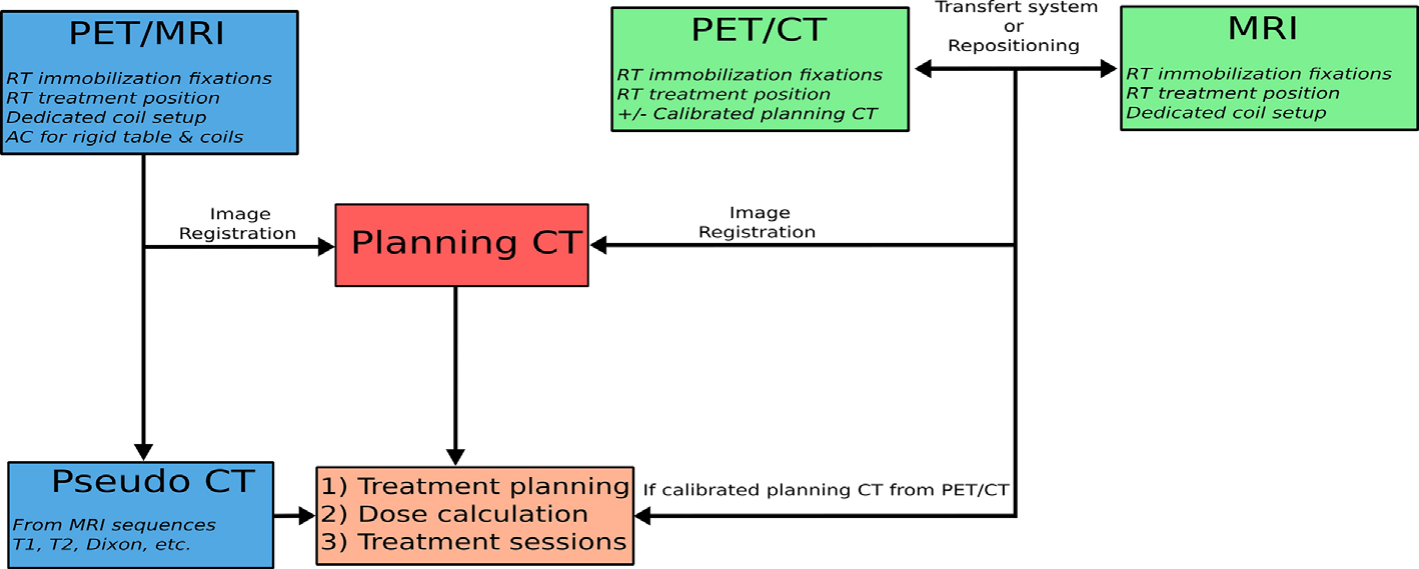
Diagram of the process for performing the PET/CT/MRI trimodality in the radiotherapy treatment position.

For the second technique, the PET/CT and MRI images are also performed in radiotherapy planning conditions, but the patient stands up between the two acquisitions. The patient is positioned on the first imager in radiotherapy treatment conditions using markers determined during planning CT acquisition and external lasers. He is then positioned in the same conditions to the second device. A summary of the methods of achieving PET/CT/MRI trimodality for radiotherapy is presented in **Figure 1**.

**Figure 2:**
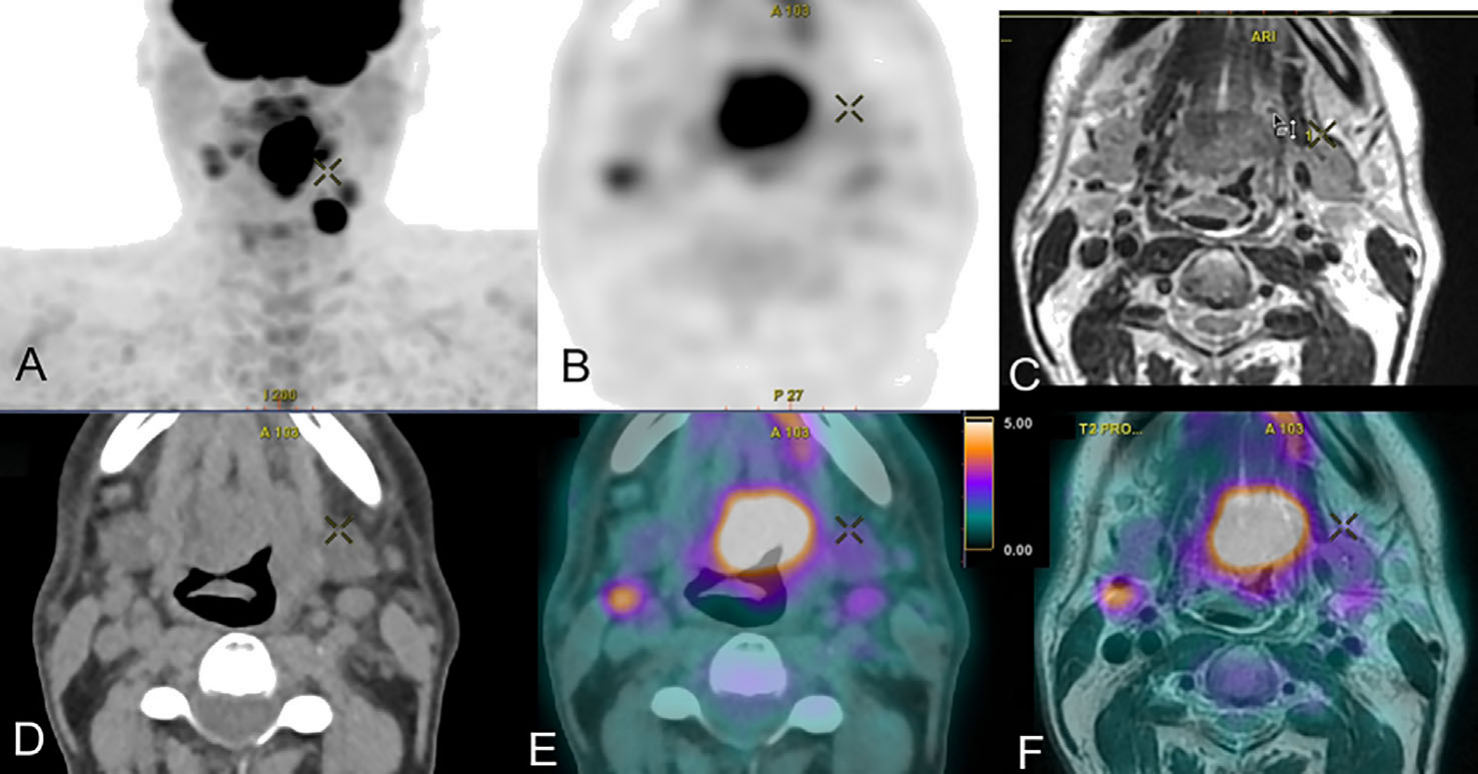
Trimodal acquisition of a cancer of the base of the tongue with in **(A)** the frontal maximum intensity projection PET image, in **(B)** the axial PET FDG acquisition, in **(C)** the axial T2 MRI acquisition, in **(D)** the axial CT acquisition, in **(E)** the axial PET/CT fusion and in **(F)** the axial PET/MRI fusion.

Image registration is the last step in the trimodality process. By placing the acquisitions in a common coordinate system, image registration allows correlating the information of each modality and thus improving clinical interpretation (see **Figure 2**). The image registration can be considered by two complementary approaches. The first one is material-based and consists in carrying out all the acquisitions under exactly the same conditions. First of all, the patient keeps the same position for each acquisition with the same radiotherapy immobilization fixations (23). The acquisition parameters will also allow obtaining the best possible alignment of the images by keeping the table height and choosing the same slice thickness, the same acquisition plane or 3D acquisitions with a large field of view (24).

This first approach facilitates the second software-based approach. The image registration is done manually by a physician or with the help of an automatic registration algorithm. In this second case, it is necessary to first evaluate the accuracy of the algorithm used. Several studies have evaluated CT-MRI or trimodality registration algorithms, either on phantom or from patient data (25–28). The average errors obtained are between 0.4 and 2 mm. Eventually, a dedicated trimodality image fusion method can be used for better target delineation (29). A visual validation remains essential before proceeding with the planning of radiotherapy treatment, since humans are capable of detecting transformations of at least 1 mm and 1° (30). Image fusion is the last step in this process of trimodality; it allows correlating the information of each modality and improving the clinical interpretation (see **Figure 2**).

The registration can however be altered by the presence of artifacts, notably dental artifacts for CT (31, 32). Concerning MRI, the presence of geometric distortions, related to the system or the patient (33), can alter the registration. Algorithms can be used to correct distortions, but the presence of residual distortions has been mentioned (34). These distortions will affect the registration as well as the resulting treatment planning, in particular the target volume coverage in the case of stereotactic treatment (35, 36). In the context of multimodality for radiotherapy, the optimization of acquisition parameters is a crucial step to facilitate image registration.

## Clinical Applications

### Characterization

Combining PET/CT/MRI could provide complementary information at baseline to assess the disease spread but also to guide biopsy and characterize the tumor to help decision making.

For glioma, many MRI sequences, notably perfusion and multiparametric and PET radiotracers, notably radiolabeled amino-acid like ^18^F-FDOPA (FDOPA) and ^18^F-FET (FET), are available (37). By their combination, it has been found that metabolic (FDOPA PET/CT) and anatomic (MRI) could aid in choosing the target to be biopsied under stereotactic conditions in tumors without MR enhancement (38). In glioblastoma, another study including multiparametric imaging with FET PET/CT and FDG PET/MRI (including diffusion and dynamic contrast enhanced perfusion) has shown that combining parameters in a multivariate model enabled patient-specific maps of recurrence probability, where FET was the most important parameter (39). Comparable results were observed in a study showing that combination of apparent diffusion coefficient (ADC) and FET was more accurate to detect glioma infiltration than standard MRI in enhancing gliomas (38); such an approach could allow risk-adapted radiotherapy planning (39). Finally, for brain metastasis, FDG PET associated with MRI could be interesting for lesion targeting of stereotactic radiation therapy in case of a previously irradiated recurrent tumor (40); place of FDOPA in this indication has yet to be evaluated.

For cervical cancer, low ADC value in MRI and high FDG SUV of the primary tumor are also predictive factors for identifying high-risk patients (41). More complex parameters with a radiomics analysis combining PET and MRI parameters have also been found interesting to improve the prognostic determination in locally advanced cervical cancer treated with chemoradiotherapy (42).

Finally, for lung cancers, combination of MRI and PET can help to determine the prognostic with poor issues when low ADC value in MRI and high FDG SUVmax in PET are associated before stereotactic body radiotherapy (43) or when parameters derived from DCE and FDG PET parameters are associated (44).

### Planning

For Head and Neck Cancers, it has been known for a long time that image fusion between FDG PET and MRI/CT is useful with regard to the determination of GTV and CTV for 3D conformal radiotherapy with preservation of normal tissues for the majority of the cases (45), with an interest to do them in treatment position, notably in case of intra-cranial tumor extension, heavy metal dental work, or contraindication of contrast enhanced CT (46). A special irradiation setup including thermoplastic mask, flat table, and head support can be used to allow a precise image co-registration of trimodality PET/CT MRI (47). As, according to pathological correlative series, no imaging modality completely encompasses the tumor, it can be useful to create a composite target volume derived from multi-modal imaging (48–51), notably for dose painting, consisting of delivering a heterogeneous irradiation within the tumor volume, as FDG PET and multiparametric MRI, in particular DWI, contain correlated but also independent information (47, 52–54). Concerning PET, other radiotracers than FDG can be used, such as FDOPA for skull base paraganglioma (55).

At the brain level, for gliomas treated with 3D conformal radiotherapy, or even by stereotactic radiotherapy by gamma-knife (9), the integration of both MRI and amino-acid PET/CT may help to improve GTV coverage by avoiding larger discrepancies between physical and biological imaging techniques (56–60), notably for the area of suspected non-enhancing tumor (61). Moreover for meningioma, ^68^Ga-DOTATOC PET, exploring the expression of SST2 receptors, can be usefully associated with CT and MRI for the treatment planning (62), notably to detect and assess the extent of infracranial meningioma invasion (63) and with an impact on sparing normal tissues (64), although all locations do not benefit from this trimodality (65).

For lung cancer, improvements in radiotherapy techniques (IMRT) make it important to manage the definition of volume and its mobility (66). The delineation of tumor lesions in lung cancer patients based on PET/CT is advisable in radiotherapy treatment planning and for locally advanced non-small cell lung cancer treated with IMRT or SRT, PET/CT being regarded as an indispensable staging procedure. Respiratory gating techniques (4D PET/CT) optimize radiotherapy of lung cancer to reduce toxicities especially the pulmonary and cardiac late toxicities (67, 68). The place of trimodality PET/CT/MRI has however to be defined in this indication.

For cervical cancer, a study on a cohort of 134 patients has shown that dose delivered to the primary cervical tumor by the combination of MRI-guided high-dose-rate PET/CT-guided IMRT brachytherapy was highly correlated with local tumor control (69). However, the delineation stays difficult as tumor volume discrepancies are observed between MRI and PET/CT GTV (70) even if it could decrease inter-observer variability (71). If FDG PET/CT appears superior as a functional imaging modality when compared with DW MRI in tumor contouring (72), a threshold around 30% of the FDG SUVmax appears to provide the best segmentation for this cancer (73).

Concerning prostatic cancer, multimodality, in particular multiparametric MRI and ^68^Ga-PSMA PET/CT, offers now large possibilities whatever the risk of the disease. In low-risk patients, selection of patients for active surveillance or treatment is improved; for intermediate-risk patients, it can help to select patients for supplemental brachytherapy; for high-risk patients it can help to guarantee adapted tumor volume segmentation, and finally, for recurrent or metastatic disease, it offers opportunities for more accurate assessment of tumor burden and treatment response (74). Therefore, it was found in a prospective study that combination of PSMA PET and multiparametric MRI provided a reliable TNM staging in patients with prostate cancer with a change in the therapeutic management for almost one third of the patients (75), PSMA PET being particularly interesting to delineate lymph node metastases (76). Moreover, for focal dose escalation to the dominant intraprostatic lesions, ^68^Ga-PSMA PET/CT and multiparametric MRI provide concordant results for delineation in nearly 50% of the lesions, with a PET GTV significantly larger than MRI GTV and which could have a role in treatment planning with intraprostatic dose escalation (77), notably because dose distribution within dominant intraprostatic lesions defined by multiparametric MRI and/or PSMA PET imaging is an independent risk factor for biochemical failure after primary external beam radiation therapy (78). If further studies are needed to confirm the optimal imaging techniques (79), it is already possible to combine multiparametric MRI and PSMA PET with higher tumor control probability with minimal to no increase of normal tissue complication probability compared to dose escalation on GTV defined on only one imaging modality (80, 81). Moreover trimodality with PSMA PET/CT/MRI can be used to orient the therapy in case of biochemical relapse after treatment (82).

For rectal cancer, trimodality PET/CT/MRI has been known to be possible for a relatively long time, notably to allow dose escalation on primary tumor (83). As a mobile organ, non-rigid registration between PET/CT and MRI shows good results, but this must be considered for the treatment planning (83). FDG PET/CT adds therefore information to MRI, with potentially a larger GTV in total when using the union of MRI and PET, and new or differently evaluated lesions in as many as 15% of the patients, potentially changing the treatment (84); further studies are necessary to well define the place of FDG PET in this indication (85, 86).

### Adaptative Radiotherapy

Functional and anatomical data can be used not only prior to treatment, but also during and after treatment to guide ART, by improving the tumor targeting while better sparing the OARs, as well as determine tumor response (87). If the ART approach has been based first historically on per-treatment CT and/or CBCT images, it is now possible with MRI-linear accelerator (MRI-linac), combining an MRI and a linear accelerator, allowing an MRI acquisition before each treatment delivery (88). PET-linac is also emerging even if it remains less mature than MRI-linac (89). While extremely promising, the utilization of functional adaptation in radiation therapy is only beginning and needs more prospective clinical validation (90).

## Perspectives

If trimodality can already be used in some indications, its usefulness remains to be confirmed. To this goal, several clinical studies are in progress. Concerning prostate cancer, our team is exploring in the ongoing DEMETER study (NCT03734757), which will include 20 patients, the interest of the association of PET/CT with ^18^F-choline and MRI compared to standard initial staging (CT, MRI and bone scan) to determine radio-therapeutic volumes. Another recently opened phase 2 study (NCT04402151), which aims to include 50 patients, will explore the interest of combination of PSMA PET/MRI to radiation delivery with a MRI-Linac. This study is based on the principle that the combination of PET PSMA and MRI allows for better delineation of intraprostatic nodules and greater diagnostic accuracy for the detection of metastatic disease. Moreover, MRI-Linac also allows adaptive radiotherapy in addition to the planning. For cervical cancer, a prospective observational study including 237 patients (NCT01992861) is exploring the role of MRI, including DCE, DWI, and spectrometry, and FDG PET performed before, during and after radiotherapy and chemotherapy. These could help to predict patient’s response to treatment and plan treatment. Finally, for head and neck cancers, our team is performing a prospective observational study with 60 inclusions planned called TRIMODAL (NCT03897166). Many questions will be explored in this study, including the comparison of the volumes determined on FDG PET/CT and on FDG PET/CT/MRI, the quality of image registration (in particular by using an air-cushion transfer system) and the use of algorithms for anthropometric measurements in MRI and CT scanners (with Dual x-ray absorptiometry as reference standard).

## Conclusion

We have shown in this brief review the ways of carrying out a trimodality PET/CT/MRI for radiotherapy and potential clinical applications. Trimodality PET/CT/MRI, combining the strengths of the techniques and limiting the respective weaknesses, reinforces the role of imaging in guiding radiation therapy. Although this multimodality is recent, it is already possible in clinical routine with a high clinical impact and good inter-observer agreement. Clinical studies are still needed to confirm its role in clinical routine.

## Author Contributions

PD, PH, PV, OV, PG, ST helped in conceptualization and project administration. PD and PH contributed to data curation. PD and PH wrote the main manuscript text and prepared the figures. All authors contributed to the article and approved the submitted version.

## Conflict of Interest

The authors declare that the research was conducted in the absence of any commercial or financial relationships that could be construed as a potential conflict of interest.
